# Detection of Changes on Parameters Related to Heart Rate Variability after Applying Current Interferential Therapy in Subjects with Non-Specific Low Back Pain

**DOI:** 10.3390/diagnostics11122175

**Published:** 2021-11-23

**Authors:** Luis Espejo-Antúnez, Carlos Fernández-Morales, María de los Ángeles Cardero-Durán, José Vicente Toledo-Marhuenda, Juan Antonio Díaz-Mancha, Manuel Albornoz-Cabello

**Affiliations:** 1 Department of Medical-Surgical Therapeutics, Faculty of Medicine and Health Sciences, University of Extremadura, Av. Elvas, s/n, 06006 Badajoz, Spain; luisea@unex.es (L.E.-A.); m.angeles.cardero@gmail.com (M.d.l.Á.C.-D.); 2 Healthcare Center Puente Real II, Av. Federico Mayor Zaragoza, s/n, 06006 Badajoz, Spain; 3 Clinic Medicine Department, University of Miguel Hernández, Avda, De la Universidad, s/n, Elche, 03202 Alicante, Spain; josetoledo@umh.es; 4 Department of Physical Therapy, Faculty of Nursing, Physical Therapy and Podiatry, University of Seville, C/Avicena, 6, 41009 Seville, Spain; jdm@us.es (J.A.D.-M.); malbornoz@us.es (M.A.-C.)

**Keywords:** electrical simulation, interferential current therapy, low back pain, autonomic nervous system, physical therapy

## Abstract

Interferential current therapy (ICT) is an electrotherapeutic intervention that combines the advantages of high permeability from middle frequency currents and efficient tissue stimulation from low frequency currents, delivering the maximum current with high tissue permeability. The aim was to evaluate the effects of ICT on heart rate variability (HRV) and on pain perception in patients with non-specific chronic low back pain (NSCLBP). In the study, 49 patients with NSCLBP were randomly divided into an experimental (EG) and a sham group (SG). All participants received a single intervention, ICT, or simulated intervention. Outcome measures including baseline (sit-down position) and postintervention (prone position) pain, heart rate (HR), time domain parameter (rMSSD), diameters of the Poincaré plot (SD1, SD2), stress score (SS), and sympathetic/parasympathetic (S/PS) ratio were investigated. In both groups, significant statistical differences were found in perceived pain and in all HRV parameters except in HRmax. Between-group comparisons showed statistically significant differences in all variables except for HRmin and HRmean in favor of the experimental group. These changes reported an increase in parasympathetic activity (rMSSD) (*p* < 0.05) and a decrease in sympathetic activity (increase in SD2 and decrease in SS) (*p* < 0.001) and perceived pain (*p* < 0.001), with a greater size effect (η2 = 0.44) in favor of the experimental group. In conclusion, a single session of ICT can shift the autonomic balance towards increase parasympathetic dominance and decrease the sympathetic dominance and intensity of pain perceived by patients with NSCLBP.

## 1. Introduction

Back pain is one of the most common health problems among the population. Specifically, non-specific chronic low back pain (NSCLBP) is the most prevalent among adults, and it often leads to functional limitations, psychological problems, low quality of life and low productivity in the workplace [1,2]. NSCLBP is defined as persistent back and sacrum pain that lasts more than 12 weeks, occurring in 85% of cases due to unknown reasons [3]. Over the last few years, the incidence of NSCLBP in young adults has increased steadily, partly due to the peak incidence in the working-age population [1,2,3,4].

Several studies suggested that non-organic signs (NOS) should be part of routine screening in chronic pain rehabilitation, to help identify patients who require thorough psychosocial evaluation and to distinguish pain from conditions mainly determined by biological factors [5,6].

In this sense, there are NOS that have been related to back pain, such as aerobic functional capacity or somatosensory and/or autonomic function. Among the latter, heart rate variability (HRV) has been considered as a noninvasive, feasible diagnostic tool that evaluates different symptoms and clinical reference signs indicative of persistent pain [7,8,9].

Specifically, detection of changes in HRV have been considered an important diagnostic measure of neurogenic homeostatic regulatory capacity in subjects with low back pain [10]. Similarly, this measure of autonomic control/balance has been previously proposed as a measure of return-to-work decision making in subjects with musculoskeletal disorders [7].

Although there is still a knowledge gap concerning the relationship between autonomic balance functioning and NSCLBP, it can be assumed that stress and pain in patients with NSCLBP cause them to be less physically active and, consequently, deconditioned. Research into stress of patients with NSCLBP has primarily been performed with HRV parameters [8,10]. The limited evidence about HRV alterations in low back pain also suggests the need of studies to investigate if HRV parameters can be used as an outcome in clinical trials aiming to investigate the effectiveness of interventions based on emotion regulation.

Evidence-based guidelines about the management of chronic low back pain highlight the importance of recognizing that chronic pain can alter the autonomic balance, with increased sympathetic activity reflected in altered HRV [5,6,7,8,9,10,11,12,13]. These findings show a solid influence of the sympathetic and parasympathetic nervous systems in pain regulation. On this matter, the mechanisms related to the influence of the autonomic nervous system are the following: (i) activation of bodily responses to stress, (ii) a likely decrease in HRV power, and (iii) reduced resilience and self-efficacy [14]. In addition, another factor of interest is gender, with differences between males and females being reported [15]. Although persistent pain and central sensitization mechanisms have been associated with increased pain severity and poor adaptation, several studies have analyzed physiological mechanisms by which pain catastrophizing maintains pain exacerbation.

In recent years, the benefits of electrical stimulation in the symptomatic management of chronic pain have been researched. Even though this approach can expand the size of the cortical receptor area and improve the sensitivity of the somatosensory system, there is no clear consensus about which is the most effective type of electric current [16]. This could be due to the variability of effects derived from the combination with other therapies (e.g., guided exercise therapy) [17,18] to the modality used (transcutaneous, percutaneous, or by implant) [19,20,21], or to the effects of electrical stimulation parameters on motor cortex signals [22]. In this sense, ICT has shown relevant clinical benefits in different pathologies, such as in low back pain (LBP) [23] or knee osteoarthritis [24], with no clear evidence reported in other body regions such as the shoulder [25]. The use of ICT has also shown changes both in electroencephalographic tests, HRV, and pain threshold in response to different conditions of ICT [26]. 

However, despite electrotherapy treatments (including ICT) being effective for NSCLBP, they are controversial, and their evidence is uncertain. This may be due to two main reasons: First, ICT has been used together with other interventions, which has limited the studies that examine its effects in isolated conditions [27]. Second, most of the studies show results of treatments targeting mainly peripheral pain generators, despite other systems that are known to be equally responsible in chronic pain processing (including NSCLBP) [28], such as autonomic balance through HRV [14].

Although these mechanisms of pain processing have been related to demographic variables [12] and to the influence of different therapeutic modalities [5], their mode of action is still not fully understood when electrotherapy procedures are applied. The aim of this study was to examine the effects caused by ICT on perceived pain and HRV in patients with NSCLBP.

## 2. Materials and Methods

### 2.1. Study Design

The present study is a randomized, single-blind, controlled trial. This study was supervised by the Institutional Ethics Committee of CEI University Hospital Virgen Macarena and Virgen del Rocio, with ethics approval number 1591-N-16, registered in ClinicalTrials.gov: NCT04483128, available at https://clinicaltrials.gov/ct2/show/results/NCT04483128?term=04483128&draw=2&rank=1 (23 July 2020) and it was completed in accordance with the Declaration of Helsinki. We followed CONSORT statements for conducting and reporting this randomized controlled trial.

### 2.2. Participants

**Figure 1:**
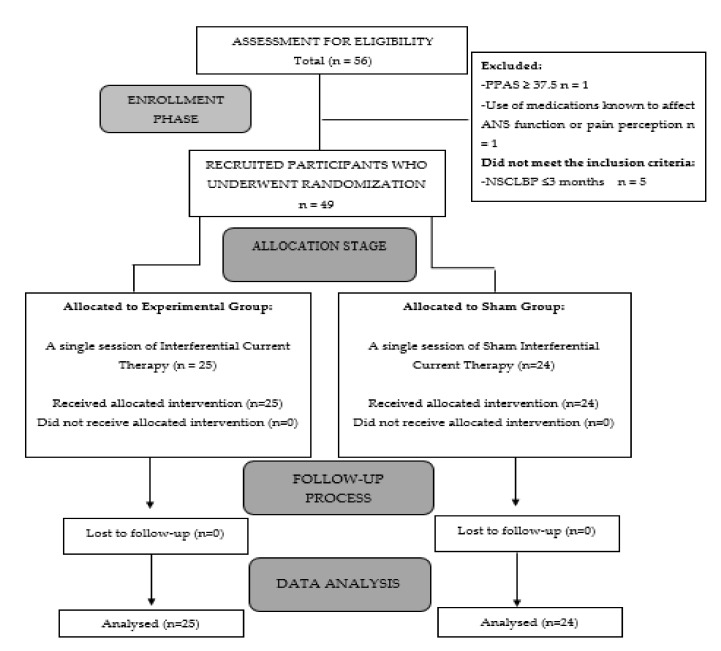
Flow-chart diagram of the progress of patients through the study phases.

Fifty-six male patients with NSCLBP, age between 19 and 65 years (M = 39; SD = 15.62) participated voluntarily in the study. The recruitment period went from 1 to 31 November 2020. The inclusion criteria were the following: (i) male subjects aged 18–65 [29]; (ii) diagnosis of NSCLBP at least 3 months ago [3]; (iii) pain intensity perceived of at least 3/10, according to the Numeric Pain Rating Scale (NPRS) [3,30]. On the other hand, the exclusion criteria were as follows: (a) any uncontrolled neurological or cardiac disorder [31]; (b) Personal Psychological Apprehension Scale (PPAS) score higher than 37.5 [32]; (c) contraindication for electrical stimulation; (d) any regular use of medications known to affect the function of the autonomic nervous system (ANS) or pain perception, including opioids, antidepressants, benzodiazepines, anti-inflammatory drugs, and beta-blockers, 2 weeks before participating in this study [14]; and (e) ineligibility to participate as determined by the researchers for other reasons. Participants were also not recruited if they had indicated they were overweight (BMI ≥ 30). Finally, a total of 49 subjects with NSCLBP met the inclusion criteria. Figure 1 provides a flow chart of the subject recruitment carried out during the study.

### 2.3. Randomization

An external website (http://www.randomization.com) (accessed on 27 May 2020) was used to complete the group allocation. Participants were randomly (using block randomization, 1:1) allocated into 1 of the 2 groups created: the experimental group (ICT) and the sham group. The randomization was performed by an external assistant. The participant had to pick up a number out of a hat. Two researchers were in charge of the study procedure. A blinded researcher collected the measurements at baseline and immediately after the treatment. The other researcher was in charge of implementing the intervention in both groups. Both were physiotherapists with more than 15 years of experience.

### 2.4. Intervention

To avoid being influenced, the participants of both groups were evaluated in separate rooms, at the same temperature, in order to maintain the environmental conditions between subjects. Group 1 (*n* = 25) received an intervention protocol using ICT (tetrapolar mode, 4 kHz carrier frequency, 65 Hz AMF and sweep at 95 Hz at 1:1 ratio) (Sonopuls 692^®^; Enraf-Nonius BV, Rotterdam, The Netherlands). The self-adhesive electrodes (Pals Platinum^©^ type, Axelgaard Manufacturing Co. Ltd., Fallbrook, CA, USA) were placed using a crossed pattern at the level of the first and fifth lumbar vertebrae, as indicated by Hurley et al. [33]. The patients had never received ICT and because of this, they were unaware of the perception. The EG were asked about perception while the control group was not because it was simulated. The application of the current produces a “pins and needles” sensation, but without visible muscle twitches. This procedure applied together with ICT has previously shown beneficial clinical effects in the same population [21]. Furthermore, the proposed ICT (low frequency and high intensity) has previously been shown to be sensitive to change in HRV measurement parameters, as well as in pain control [26]. On the other hand, group 2 (*n* = 24) received the sham intervention. Likewise, the subject was unaware of which group he belonged to in the study.

HRV was recorded in both groups in a lay prone position in the early morning after fasting overnight [19,20]. Then, HRV was recorded in the EG during the reception of ICT in the lower back (for 25 min) as proposed by previous studies [19,20]. Likewise, HRV was recorded in the SG during a sham intervention (without application of electric current) in the same corporal region. The intervention lasted 25 min, with the recording of the entire procedure lasting 35 min. Due to ethical reasons, both groups were instructed at the end of the intervention to follow a home therapeutic exercise program as reported by Bodes-Pardo et al. [34]. The follow-up by telephone was carried out after 2 weeks by the same physiotherapist. The Bioethics Committee of the University of Seville (Spain) granted ethical approval. All procedures were conducted according to the Declaration of Helsinki. Those who agreed to participate in this study provided a written informed consent.

### 2.5. Outcomes Measures

All instruments were applied in a single day and baseline and post-treatment testing times were the same for all participants. The data were collected in the same room in which the procedure was performed. The rater, who was blinded to group allocation, collected the baseline clinical data. Demographic, anthropometric, and clinical data were collected using a self-assessment questionnaire created for this study. The primary outcome was pain intensity perceived, measured according to the Numeric Pain Rating Scale (NPRS), which exhibited a standard error of measurement (SEM) of 1.02 points, corresponding to a minimum variation in the 95% confidence level (MDC_95)_ of 2 points [35]. A long-term HRV record (30 min) was simultaneously registered (5 min before the start and during the full intervention, which lasted 25 min). The secondary outcome (covariable) was the level of disability provoked by NSCLBP. In order to assess this covariable, we used the Spanish version of Roland–Morris Disability Questionnaire (RMQ) (0–24, with 0 as no disability and 24 as maximum disability). This questionnaire showed a good internal consistency (Cronbach α = 0.83) and an intraclass correlation coefficient (ICC) of 0.874 [36].

Recommendations of the Task Force of the European Society of Cardiology and the North American Society of Pacing and Electrophysiology [31], as well as instructions derived from previous studies that used the heart rate monitor Firstbeat Bodyguard^®^, were followed [19,20].

**Figure 2:**
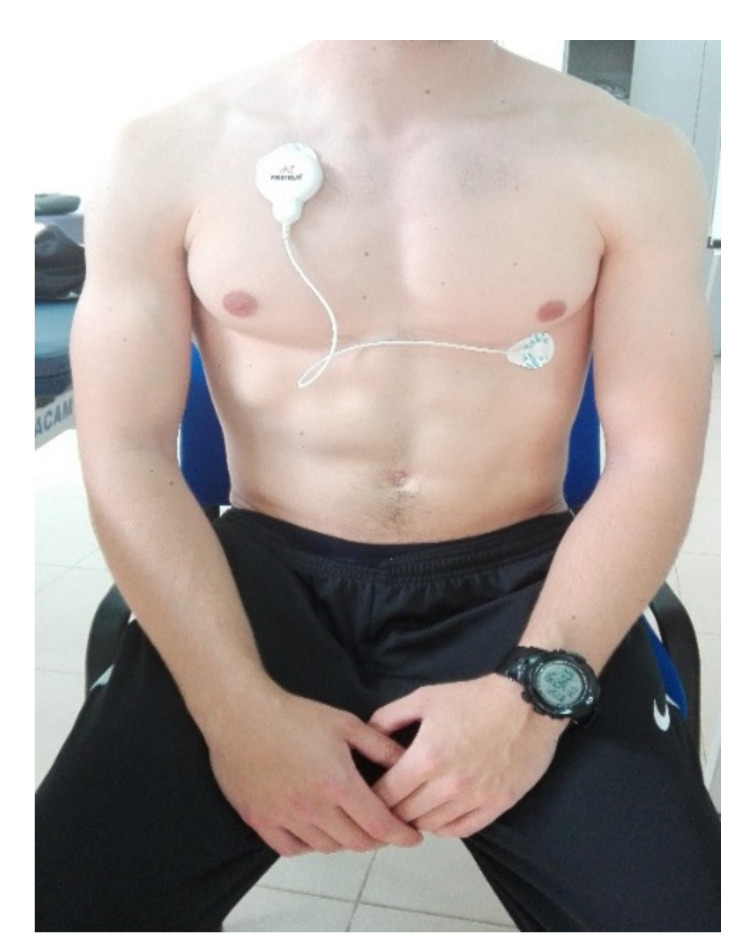
Procedure for adjusting the device before the beginning of the intervention.

The HRV R-R heartbeat interval was used as measure of autonomic modulation, which was estimated with a Firstbeat Bodyguard^®^ monitor (Firstbeat Technologies, Jyväskylä, Finland) (Figure 2). This device was used to record HRV data for 30 min (at rest and during the intervention). Data were downloaded from the device to a computer using Firstbeat Uploader software (Firstbeat Technologies, Jyväskylä, Finland). All RRI series were imported into Kubios^®^ HRV software (v.2.1.) (University of Eastern Finland, Kuopio, Finland) [37].

The HRV method is commonly used to calculate the autonomic balance is based on the Poincaré plot [38]. It has demonstrated to be extremely valid and capable of registering non-linear trends that are often presented in variation registries in the interval of time between beats (R-R) [38,39]. HRV has been validated as an accurate tool to assess the status of the autonomous nervous system (both sympathetic and parasympathetic components) under different conditions, including NSCLBP [10,40].

We also considered a checklist regarding the use of HRV collection and analysis methodology to improve the reporting of evaluation and intervention results using HRV [41]. Physiologically, we measured the mean heart rate and time domain parameters: root mean square of successive differences (rMSSD); diameters of Poincaré plot, the short-term variability’s sensitivity of HRV non-linear specter (SD1); and the long-term variability of HRV non-linear specter (SD2). SD1 is considered an indicator of parasympathetic activity [41]. The physiological meaning of SD2 is not clear, but it is thought to reflect the long-term changes in RRIs, and it is considered an inverse indicator of sympathetic activity [38]. Naranjo-Orellana et al. [42] described two new indexes to simplify the physiological interpretation of Poincaré plot: the stress score (SS) and sympathetic-parasympathetic ratio (S/PS). The SS is expressed as the inverse of SD2 diameter multiplied by 1000, and it is directly proportional to sympathetic activity at the sinus node. The S/PS ratio is expressed as the quotient of SS and SD1, and it is considered to reflect autonomic balance, that is, the relationship between sympathetic and parasympathetic activity.

### 2.6. Sample Size Calculation

The sample size was estimated using GPower 3.1.3. software (Düsseldorf, Germany). At the beginning of the study, the sample size was of 50 patients with NSCLBP. For ANOVA F-test, a total sample size of 46 participants was estimated, bearing in mind repeated measures, within-between interaction, and assuming an effect size (f) of 0.25, an alpha level of 0.05, and 90% power. The sample was inflated by 10% to account for potential dropouts, resulting in a sample size of 49 participants.

### 2.7. Statistical Analysis

Statistical analyses were carried out by an assessor blinded to the treatment allocation, using SPSS statistical software (SPSS Inc., Chicago, IL, USA), in its 21.0 version. Firstly, the normal distribution of variables was verified by the Kolmogorov–Smirnov test, after a descriptive analysis. The homogeneity of variances was observed by Levene’s test. Linearity was evaluated by bivariate scatter plots of observed residual values against the expected values. Comparisons between groups were conducted for baseline demographic and clinical data using Student’s *t*-test for continuous data and Chi-square test for categorical data. The interclass correlation coefficient (ICC) and SEM values were used to determine the reliability of the measurements. Differences in the outcome measures were detected using a repeated measures analysis of variance (ANOVA), with the group (sham group vs. experimental group) as the between-subjects factor, and time effects (baseline vs. intervention) as the within-subjects factor. Eta square (η^2^) was used to calculate the effect size (small when 0.01 ≤ η^2^ ≤ 0.06; medium when 0.06 ≤ η^2^ > 0.14; large when η^2^ > 0.14) [43]. A *p*-value < 0.05 was considered statistically significant.

## 3. Results

There were no significant differences between groups in any demographic variable. Moreover, no significant differences between EG and SG in any HRV parameters (*p* > 0.05) were found (Table 1).

Although the HRV has been validated as an accurate tool to assess the status of the autonomous nervous system, the reliability results for the present study were as follows: the ICC and standard error measurement (SEM) were calculated in the EG for NPRS = 0.58 (0.18–0.82), SEM: 1.35; min heart rate = 0.93 (0.83–0.97), SEM: 0.33; max heart rate = 0.70 (0.31–0.87), SEM: 0.79; mean heart rate = 0.91 (0.8–0.96), SEM: 0.38; rMSSD = 0.96 (0.9–0.98), SEM: 0.32; SD1 = 0.82 (0.59–0.92), SEM: 2.29; SD2 = 0.36 (−0.44–0.72), SEM: 15.96; SS = 0.23 (−0.77–0.67), SEM: 3.03; and S/PS ratio = 0.67 (0.1–0.79), SEM: 0.12.

Table 2 shows the baseline and post-intervention scores and the mean differences between and within groups for perceived pain and heart rate variability parameters. Compared with baseline values, SG only showed a statistically significant increase in SD1 (*p* = 0.007, d = 0.34) and in SS (*p* < 0.001, d = 0.30) and a decrease in SD2 (*p* < 0.001, d = 0.31) and S/PS ratio (*p* = 0.003, d = 0.38) after intervention. In comparison with baseline values, the EG exhibited statistically significant increase in rMSSD (*p* < 0.001, d = 0.55), SD1 (*p* < 0.001, d = 0.52) and SD2 (*p* < 0.001, d = 0.47), whereas it showed a significant decrease in SS (*p* = 0.016, d = 0.28) and S/PS ratio (*p* < 0.001, d = 0.54) after intervention. Moreover, Table 2 includes a between-group comparison, which showed statistically significant differences in NPRS, rMSSD, SD1, SD2, SS, and S/PS ratio values in favor of the experimental group (Table 2). Although there were differences in HRMax, the lack of statistically significant differences for HRmin and HRmean means that this should not be taken into account from a methodological and clinical point of view.

Analysis of variance for repeated measures within the group (sham group vs. experimental group) like the between-subjects factor, and time effects (baseline vs. intervention) like the within-subjects factor showed statistically significant differences, which were found to favor the EG in the intensity of pain perceived (NPRS) at F_1, 47_= 37.28 (*p* < 0.001) η2 = 0.44 and the following HRV parameters: rMSSD, F_1, 47_ = 6.73 (*p*= 0.013) and η2 = 0.13; SD2, F_1, 47_ = 20.40 (*p* < 0.001) and η2 = 0.30; SS, F_1, 47_ = 16.17 (*p* < 0.001) and η2 = 0.26; and Max HR, F_1, 47_ = 6.01 (*p* = 0.018) and η2= 0.11. The parameters with non-statistical differences were as follows: Min HR, F_1, 47_ = 0.058 (*p*= 0.811) and η2 = 0.001; mean HR, F_1, 47_ = 0.97 (*p*= 0.329) and η2 = 0.02; SD1, F_1, 47_ = 1.62 (*p*= 0.209) and η2 = 0.03; and S/PS ratio, F_1, 47_ = 2.78 (*p*= 0.102) and η2 = 0.05.

Finally, these changes reported an increase in parasympathetic activity (rMSSD) (*p* < 0.05) and a decrease in sympathetic activity (increase in SD2 and decrease in SS) in favor of the experimental group. Correlation analysis showed no statistically significant association between rMSSD and age, BMI, NPRS, RMQ, and PPAS, respectively, both at baseline and at the end of the intervention for the whole sample. A statistically significant correlation (Spearman’s Rho) was obtained between rMSSD and perceived pain (NPRS) after the intervention for the whole sample (R = −0.625; *p* < 0.001). Similarly, a statistically significant correlation (Spearman’s Rho) was reported for the experimental group between rMSSD and NPRS (R = −0.468; *p* = 0.021) and between SD1 and NPRS (R = −0.495; *p* = 0.014) at baseline. At the end of the study, a significant correlation (Spearman’s Rho) was also obtained between rMSSD and NPRS (R = −0.458; *p* = 0.024) and SD2 and NPRS (R = −0.695; *p* < 0.001).

**Figure 3:**
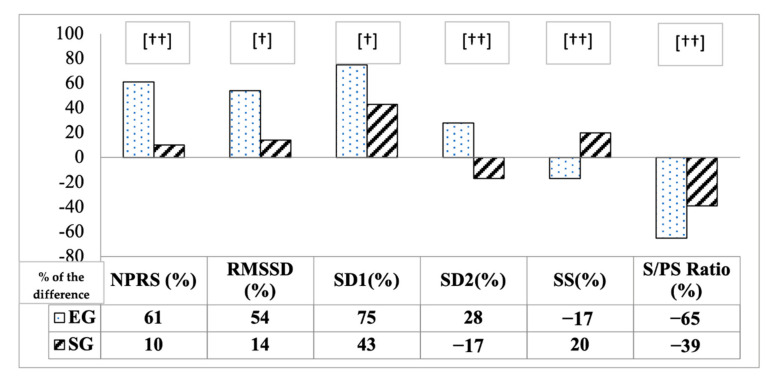
Percentage differences observed in pain intensity and HRV parameters between groups after intervention (independent samples *t*-test; [†]: *p* < 0.05; [††]: *p* < 0.001).

Figure 3 shows the statistically significant differences between both groups for NPRS (61% vs. 10%) and for the different HRV parameters (by independent samples *t*-test), highlighting the differences expressed in percentage observed in the parameters that reflect in parasympathetic activity (rMSSD: 54% vs. 14% and SD1: 75% vs. 43%), as well as in S/PS ratio (−65% vs. 39%).

## 4. Discussion

The aim of this study was to examine the effects of a single intervention of interferential current therapy (ICT) on pain perception and heart rate variability (HRV) parameters of subjects diagnosed with non-specific low back pain (NSCLPB). The findings are consistent with previous studies in suggesting the immediate influence of electrical stimulation applied on the spinal column, regarding the perceived pain intensity [17,23,26,44] and HRV [10,20,22,45].

As for perceived pain intensity, our results showed statistically significant differences between both groups, with differences in EG also appearing (4.54 points; change: 60.5%) above the minimum clinical important change for the numeric pain rating scale (NPRS) [29]. The influence of the electrical current on pain relief has been reported for the effects on the endogenous inhibitory analgesic mechanisms (involved in the SNC and SNA), modifying the sensitive and emotional perception of pain [46].

In particular, ICT is an intervention method that combines the advantages of high permeability from middle frequency currents and efficient tissue stimulation from low frequency currents, delivering the maximum current with high tissue permeability. Therefore, changes observed on the deep back muscles could be due to the characteristics of the current used, being consistent with previous studies developed with LBP patients, in which effects on variables controlled by nociceptive pathways were associated (e.g., pain perceived with the Visual Analogue Scale, VAS) [26] with a decrease in variables regulated by the autonomic nervous system (e.g., heart rate) [47,48]. Wolff et al. [47] explained this relationship by the interaction of the lower paraspinal muscle tension with central sensitization mechanisms. On this matter, recent studies have associated the effects on perceived pain intensity with parameters related to HRV after using non-invasive physiotherapeutic treatments. Abuín-Porras et al. [48] observed a decrease in perceived pain (dif vas: 1.8; change: 24.7%) and a significative increase in parasympathetic activity (rMSSD, min HR, mean HR) after a session of manual therapy in the lumbopelvic area of men with NSCLBP. In our study, the ICT led to a decrease in perceived pain (dif vas: 4.54; change: 60.5%).

Changes observed in pain intensity perceived in SG could be explained by the placebo component that showed applying electrotherapy [49]. However, this fact is not certain due to the existence of contradictory results. Our results differ from the ones shown by Franco et al. [17,18]. In one of their studies, they indicate that the application of ICT before performing therapeutic exercise, such as pilates, is not more effective than ICT placebo [46]. Whereas, in a recent study, they indicate that the group receiving ICT presented a pain reduction of 30% in just one session, 50% in two sessions, and pain remission after three sessions faster than the placebo group [18]. Considering this, it appears that a sequence in which ICT is combined with therapeutic exercise could be the key to the reported benefits. The inclusion in our study of a therapeutic exercise program before ICT or placebo ICT intervention could have an influence on patient education and therapeutic alliance (defined as a positive connection between the patient and the therapist), as well as on the reduction of pain sensitivity reported by the subjects [50].

On the other hand, HRV was also analyzed after electric stimulation, showing increases in parasympathetic activity (rMSSD and SD1) and decreases in the sympathetic domain (SD2) [42]. Changes observed after applying ICT on the spinal column could be due to the following: (i) the effects generated on the spinal cord, stimulating downstream inhibitory systems; and (ii) the effects derived from these systems on the restoration of autonomic imbalance [44].

The results showed statistically significant increases in rMSSD only in the EG. These results are consistent with the ones obtained by Abuín-Porras et al. [48] after applying a session of manual therapy (36% of change), where the change percentage was lower than the one obtained in the present study (54% of change). According to Koening et al. [12], changes in rMSSD in subjects with chronic pain are mainly due to pain perception. When comparing both groups, statistically significant differences were also observed, with reported differences up to 40% of change (Figure 3). This parameter indirectly shows the vagal and emotional activity and, therefore, the intervention’s influence on downstream inhibitory systems.

SD1 showed a statistically significant increase in both groups, between the beginning and the end of the intervention (SG: 43%; EG: Diff 75%), reporting statistical differences between both groups, in favor of EG (Diff: 32%), with a moderate effect size (d = 0.52). Changes observed in both groups could be influenced by the differences of the subject position, who were sitting in the baseline state and in prone position during the intervention. The position’s influence on autonomic balance recordings has already been reported, where a sympathetic dominance exists in the sitting position here as, in young and healthy men, the dominance lies in the prone position [48,51,52]. These arguments could explain the lack of interaction between group and time in the analysis of the variance.

In regard with SD2, a statistically significant decrease in sympathetic activity was observed in EG, whereas the SG showed a statistically significant increase (Table 2). These statistically significant differences were also established between groups, reporting an overall difference in the sympathetic activity decrease between groups of 45%, in favor of EG. The SS parameter’s difference between both groups was also statistically significant, but with opposite trends. Whereas in the EG, a statistically significant increase is shown in this parameter, the SG reported a decrease in it, with statistically significant differences between both groups, with a difference of 37% in favor of the EG. These results indicate the intervention’s impact via ICT on HRV, ensuring that sympathetic activity does not generate an autonomic imbalance that could lead to an increase in the pain perceived [43].

Lastly, the S/PS ratio also showed changes after ICT in the autonomic balance (it reduces sympathetic activity, prevailing parasympathetic activity), observing statistically significant differences between groups (26% greater reduction in favor of the EG) (Figure 3). A S/PS ratio of ≥0.3 at rest can indicate whether an excess of sympathetic activity or lack of recovery in parasympathetic activity is present [33]. In both groups, baseline measures are observed above 0.3, indicating an excess of sympathetic activity. After applying the intervention with the subjects in the prone position, normal values in the EG were achieved (0.31). Despite this, it failed to come within the normal value for reaching autonomic balance in the EG. Although no statistically significant interaction was shown in the repeated measures analysis between groups, the EG came very close to normal values. This could again be due to the fact that the position of the patient could influence the effects achieved and is therefore estimated to be less than that observed (as in SD1, these arguments could explain the lack of interaction between groups and time in the analysis of the variance).

### Clinical Implications

HRV can be proposed as an indirect measure of vagal activity, and perhaps a reflection of the function of downstream endogenous inhibitory systems [15] could allow it to be used, in a clinical context, as an indicator in subjects with chronic pain follow-up. The regulatory effects observed in the long term on autonomous balance could allow for ICT to be used as supplementary mechanism for the inhibition of sympathetic activity in subjects with NSCLBP, as suggested by Tousignant-Laflammeet et al. [15] regarding electrical stimulation. The results obtained, together with the lack of clear evidence about which procedure is the most appropriate (transcutaneous vs. percutaneous), [33] allow for decisions to be made regarding which modality is more appropriate for each patient.

The main limitations of the study were that the intervention was carried out in a single session and exclusively to males, which could limit the effects observed as well as the difficulty to generalize the results to female patients. as Additionally, the lack of measurement of psychosocial factors associated with persistent pain was a limitation, which could also influence HRV. Furthermore, the sensation (pins and needles) might indicate that the patients would associate this with a therapy. Likewise, the absence of any sensation during the experiment in the prone position could lead to outcome bias. In order to preserve the blinding of the sham group, future studies would benefit from applying a sham intervention that imitates the experimentation intervention. Finally, further studies are needed to determine whether there is an association between these variables and the autonomic balance of subjects with persistent pain.

Based on the results observed, HRV can be considered as a factor providing diagnosis for NOS in disorders characterized by persistent pain [8,10], and future studies are needed to report the behavior of these markers when comparing healthy and diseased subjects.

## 5. Conclusions

A single session of ICT can shift the autonomic balance towards parasympathetic dominance and decrease the sympathetic dominance and intensity of pain perceived in patients with NSCLBP. This is the first study to show that using ICT as diagnostic measure and therapy, respectively, in subjects with NSCLBP can lead to an improvement of the autonomic balance.

## Figures and Tables

**Table 1:** Baseline characteristics of the participants.

	Total Sample (*n* = 49)	Experimental Group (*n* = 25)	Sham Group (*n* = 24)	*p-*Value *
Age (years)	39 (15.62)	37 (16.56)	40 (14.96)	0.56
Height (cm)	177.49 (5.96)	177.46 (5.81)	177.52 (6.21)	0.97
Weight (kg)	82.29 (14.52)	81.18 (11.07)	83.36 (17.37)	0.61
BMI	25.27 (2.95)	25.21 (2.79)	25.33 (3.15)	0.88
PPAS	24 (5.91)	24 (5.09)	24 (6.70)	0.98
NPRS	7.37 (1.07)	7.50 (1.18)	7.24 (0.97)	0.40
RMQ	12.31 (4.36)	12.63 (4.39)	12.00 (4.41)	0.62
Min HR (bmp)	66.13 (12.86)	67.31 (13.74)	65.00 (12.14)	0.53
Max HR (bmp)	88.90 (15.26)	84.64 (13.31)	92.98 (16.14)	0.06
Mean HR (bmp)	75.00 (13.05)	73.81 (13.16)	76.14 (13.11)	0.54
rMSSD (ms)	31.56 (13.03)	33.87 (9.16)	29.34 (15.77)	0.23
SD1 (ms)	32.57 (24.01)	33.53 (24.40)	31.64 (24.10)	0.79
SD2 (ms)	54.82 (13.61)	57.13 (11.73)	52.63 (15.10)	0.25
SS (ms)	19.46 (5.27)	18.19 (3.59)	20.67 (6.33)	0.10
S/PS Ratio	0.92 (0.59)	0.88 (0.60)	0.95 (0.60)	0.66

Data are reported as mean (SD). BMI: body mass index; PPAS: Personal Psychological Apprehension Scale; NPRS: Numeric Pain Rating Scale; RMQ: Roland–Morris Questionnaire; Mean HR = average heart rate, beats per minute (bpm); SD1 = transverse axis of Poincaré plot millisecond (ms); SD2 = longitudinal axis of Poincaré plot; SS = stress score (inverse of diameter SD2 × 1000); S/PS ratio = quotient of SS and SD1. * One-way ANOVA. Statistically significant differences (*p* < 0.05).

**Table 2:** Baseline, post-intervention and mean score changes of HRV parameters.

Variable	Group	Baseline	Intervention	Within-Group Mean Changes	*d*	Between-Group Mean Changes
NPRS	SG EG	7.24 (0.97) 7.50 (1.18)	6.52 (1.12) 2.96 (1.04)	0.72 [0.37/1.06] ** 4.54 [4.03/5.05] **	0.32 0.89	3.56 [2.93/4.18] ^††^
HR Min (bpm)	SG EG	65.00 (12.14) 67.31 (13.74)	61.37 (11.55) 60.67 (10.41)	3.62 [1.91/5.34] ** 6.64 [3.98/9.29] **	0.15 0.26	0.69 [−7.03/5.63]
HR Max (bpm)	SG EG	92.98 (16.14) 84.64 (13.31)	92.71 (16.69) 80.94 (18.45)	0.27 [−5.99/6.53] 3.70 [−2.80/10.21]	- -	11.77 [1.67/21.88] ^†^
HR Mean (bpm)	SG EG	76.14 (13.11) 73.81 (13.16)	72.55 (14.03) 67.81 (11.54)	3.59 [0.79/6.38] * 5.99 [3.05/8.94] **	0.13 0.24	4.73 [2.66/12.13]
rMSSD (ms)	SG EG	29.34 (15.77) 33.87 (9.16)	33.59 (21.84) 55.21 (17.18)	4.24 [0.46/8.95] 18.33 [13.24/23.43] **	- 0.55	18.62 [7.29/29.95] ^†^
SD 1 (ms)	SG EG	31.64 (24.10) 31.46 (20.54)	45.37 (11.36) 57.15 (21.73)	13.72 [4.07/23.37] * 23.61 [18.21/29.02] **	0.34 0.52	11.77 [1.86/21.68] ^†^
SD 2 (ms)	SG EG	52.63 (15.10) 57.13 (11.73)	43.52 (12.76) 73.19 (17.72)	9.10 [5.00/13.21] ** 16.06 [10.41/21.69] **	0.31 0.47	29.67 [20.85/38.48] ^††^
SS (ms)	SG EG	20.67 (6.33) 18.20 (3.59)	24.80 (6.71) 15.02 (6.70)	4.13 [2.07/6.18] ** 3.17 [0.65/5.69] *	0.30 0.28	9.77 [5.91/13.63] ^††^
S/PS Ratio	SG EG	0.95 (0.60) 0.88 (0.60)	0.58 (0.23) 0.31 (0.20)	0.37 [0.14/0.60] * 0.57 [0.35/0.78] **	0.38 0.54	0.27 [0.14/0.39] ^††^

Data are reported as mean (SD) or 95% confidence level. *d* = effect size (d’ Cohen). Interventions in the sham group (SG) and experimental group (EG) consisted in a IFC intervention without and with current, respectively. Mean HR = average heart rate, beats per minute (bpm); SD1 = transversal axis of Poincaré plot, millisecond (ms); SD2 = longitudinal axis of Poincaré plot; SS = stress score (inverse of diameter SD2 × 1000); S/PS ratio = quotient of SS and SD1. * Paired samples *t*-test indicates statistically significance within-group differences (*p* < 0.05) ** Paired samples *t*-test indicates statistically significance within-group differences (*p* < 0.001) ^†^ Independent Samples *t*-test. Indicates statistically significance between-group differences (*p* < 0.05) ^††^ Independent samples *t*-test indicates statistically significance between-group differences (*p* < 0.001).

## References

[1] Hoy D., Bain C., William G., March L., Brooks P., Blyth F., Woolf A., Vos T., Buchbinder R. (2012). A systematic review of the global prevalence of low back pain. Arthritis Rheum..

[2] Nambi G.S., Inbasekaran D., Khuman R., Surbala D., Devi S., Jagannathan K. (2014). Changes in pain intensity and health related quality of life with Iyengar yoga in nonspecific chronic low back pain: A randomized controlled study. Int. J. Yoga.

[3] Alsufiany M.B., Lohman E.B., Daher N.S., Gang G.R., Shallan A.I., Jaber H.M. (2020). Non-specific chronic low back pain and physical activity: A comparison of postural control and hip muscle isometric strength: A cross-sectional study. Medicine.

[4] Becker A., Held H., Redaelli M., Strauch K., Chenot J.F., Leonhardt C., Keller S., Baum E., Pfingsten M., Hildebrandt J. (2010). Low back pain in primary care: Cost of care and prediction of future health utilization. Spine.

[5] Oesch P., Meyer K., Jansen B., Kool J. (2015). Functional capacity evaluation: Performance of patients with chronic non-specific low back pain without Waddell signs. J. Occup. Rehabil..

[6] Waddell G., McCulloch J.A., Kummel E., Venner R.M. (1980). Nonorganic physical signs in low-back pain. Spine.

[7] Ansuategui Echeita J., van der Wurff P., Killen V., Dijkhof M., Grootenboer F., Reneman M. (2020). Lifting capacity is associated with central sensitization and non-organic signs in patients with chronic back pain. Disabil. Rehabil..

[8] McCraty R., Atkinson M., Tomasino D., Bradley R.T. (2009). The Coherent Heart: Heartbrain Interactions, Psychophysiological Coherence, and the Emergence of System-Wide Order.

[9] Echeita J.A., Preuper H.R.S., Dekker R., Stuive I., Timmerman H., Wolff A.P., Reneman M.F. (2020). Central Sensitisation and functioning in patients with chronic low back pain: Protocol for a cross-sectional and cohort study. BMJ Open.

[10] Bandeira P., Reis F., Sequeira V., Chaves A., Fernandes O., Arruda-Sanchez T. (2021). Heart rate variability in patients with low back pain: A systematic review. Scand. J. Pain.

[11] Telles S., Sharma S.K., Gupta R.K., Bhardwaj A.K., Ballkrishna A. (2016). Heart rate variability in chronic low back pain patients randomized to yoga or standard care. BMC Complement. Altern. Med..

[12] Koening J., Loerbroks A., Jarczok M.N., Fischer J.E., Thayer J.F. (2016). Chronic Pain and Heart Rate Variability in a Cross-Sectional Occupational Sample: Evidence for Impaired Vagal Control. Clin. J. Pain.

[13] Tracy L.M., Ioannou L., Baker K.S., Gibson S.J., Geogriou-Karistianis N., Giummarra M.J. (2016). Meta-analytic evidence for decreased heart rate variability in chronic pain implicating parasympathetic nervous system dysregulation. Pain.

[14] Berry M.E., Chapple I.T., Ginsbeg J.P., Gleichauf K.J., Meyer J.A., Nagpal M.L. (2014). Non-pharmacological Intervention for Chronic Pain in Veterans: APilot Study of Heart Rate Variability Biofeedback. Glob. Adv. Health Med..

[15] Tousignant-Laflamme Y., Marchand S. (2006). Sex differences in cardiac and autonomic response to clinical and experimental pain in LBP patients. Eur. J. Pain.

[16] Almeida C.C., Silva V.Z.M.D., Júnior G.C., Liebano R.E., Durigan J.L.Q. (2018). Transcutaneous electrical nerve stimulation and interferential current demonstrate similar effects in relieving acute and chronic pain: A systematic review with meta-analysis. Braz. J. Phys. Ther..

[17] Franco Y.R., Franco K.M., Silva L.A., Silva M.O., Rodríguez M.N., Liebano R.E., Cabral C.N. (2018). Does the use of interferential current prior to pilates exercises accelerate improvement of chronic nonspecific low back pain?. Pain Manag..

[18] Franco K.M., Franco Y.R., de Oliveira N.B., Miyamoto C., Oliveira Santos M., Liebano R.E., Cabral C.N. (2017). Is Interferential Current Before Pilates Exercises More Effective Than Placebo in Patients With Chronic Nonspecific Low Back Pain? A Randomized Controlled Trial. Arch. Phys. Med. Rehabil..

[19] De la Cruz-Torres B., Albornoz-Cabello M., García-Bermejo P., Naranjo-Orellana J. (2016). Autonomic responses to ultrasound-guided percutaneous needle electrolysis of the patellar tendon in healthy male footballers. Acupunct. Med..

[20] García-Bermejo P., De La Cruz-Torres B., Naranjo-Orellana J., Albornoz-Cabello M. (2018). Autonomic Responses to Ultrasound-Guided Percutaneous Needle Electrolysis; Effect of Needle Puncture or Electrical Current?. J. Altern. Complement. Med..

[21] Meglio M., Cioni B., Rossi G.F., Sandric S., Santarelli P. (1986). Spinal cord stimulation affects the central mechanisms of regulation of heart rate. Appl. Neurosurg..

[22] Sung-Hyoun C., Seon-Chil K. (2020). Changes in Electroencephalography by Modulation of Interferential Current Stimulation. Appl. Sci..

[23] Albornoz-Cabello M., Maya-Martín J., Domínguez-Maldonado G., Espejo-Antúnez L., Heredia-Rizo A.M. (2017). Effect on interferential current therapy on pain perception and disability level in subjects with chronic low back pain: A randomized controlled trial. Clin. Rehabil..

[24] Alqualo-Costa R., Thomé G.R., Perracini M.R., Liebano R.E. (2018). Low-level laser therapy and interferential current in patients with knee osteoarthritis: A randomized controlled trial protocol. Pain Manag..

[25] Nazligul T., Akpinar P., Aktas I., UnluOzkan F., CagliyanHarteyoglu H. (2018). The effect of interferential current therapy on patients with subacromial impingement syndrome: A randomized, double-blind, sham-controlled study. Eur. J. Phys. Rehabil. Med..

[26] Sung-Hyoun C. (2019). Frequency and Intensity of Electrical Stimulation of Human Sympathetic Ganglia Affect Heart Rate Variability and Pain Threshold. Appl. Sci..

[27] Fuentes J.P., Armijo-Olivo S., Magee D.J., Gross D.P. (2010). Effectiveness of interferential current therapy in the management of musculoskeletal pain: A systematic review and meta-analysis. Phys. Ther..

[28] Woolf C.J. (2011). Central sensitization: Implications for the diagnosis and treatment of pain. Pain.

[29] Koening J., Thayer J.F. (2016). Sex differences in healthy human heart rate variability: A meta-anaylsis. Neurosci. Biobehav. Rev..

[30] Dworkin R.H., Turk D.C., Farrar J.T., Haythornthwaite J.A., Jensen M.P., Katz N.P., Kerns R.D., Stucki G., Allen R.R., Bellamy N. (2005). Core outcome measures for chronic pain clinical trials: IMMPACT recommendations. Pain.

[31] Camm A.J. (1996). Heart rate variability. Standards of measurement, physiological interpretation and clinical use. Task Force of the European Society of Cardiology and the North American Society of Pacing and Electrophysiology. Eur. Heart J..

[32] Albornoz M., Rebollo J., García R. (2005). Escala de Aprensión Psicológica Personal (EAPP) en Fisioterapia. Rev. Iberoam. Fisioter. Y Kinesiol..

[33] Hurley D.A., Minder P.M., McDonough S.M., Walsh D.M., Moore A.P., Baxter D.G. (2001). Interferential therapy electrode placement technique in acute low back pain: A preliminary investigation. Arch. Phys. Med. Rehabil..

[34] Bodes-Pardo G., Lluch-Girbés E., Roussel N., Gallego-Izquierdo T., Jiménez-Penick V., Pecos-Martín D. (2018). Pain Neurophysiology Education and Therapeutic Exercise for Patients with Chronic Low Back Pain: A Single-Blind Randomized Controlled Trial. Arch. Phys. Med. Rehabil..

[35] Childs J.D., Piva S.R., Fritz J.M. (2005). Responsiveness of the Numeric Pain Rating Scale in Patients with Low Back Pain. Spine.

[36] Kovacs F.M., Llobera J., Gil del Real M.T., Abraira V., Gestoso M., Fernández C., Kovacs-Atención Primaria Group (2002). Validation of the spanish version of the Roland-Morris questionnaire. Spine.

[37] Ramírez-Adrados A., Beltrán-Velasco A.I., González de Ramos C., Fernández-Martínez S., Martínez-Pascual B., Fernández-Elías V.E., Clemente-Suárez V.J. (2020). The effect of final dissertation defense language, native vs. non-native, in the psychophysiological stress response of university students. Physiol. Behav..

[38] Mourot L., Bouhaddi M., Perrey S., Rouillon J.D., Regnard J. (2004). Quantitative Poincaré plot analysis of heart rate variability: Effect of endurance training. Eur. J. Appl. Physiol..

[39] Brennan M., Palaniswami M., Kamen P. (2001). Do existing measures of Poincaré plot geometry reflect nonlinear features of heart rate variability?. IEEE Trans. Biomed. Eng..

[40] Catai A.M., Pastre C.M., Fernades de Godoy M.F., Da Silva E.D., Takahashi A.C.M., Vanderlei L.C.M. (2020). Heart rate variability: Are you using it properly? Standardisation checklist of procedures. Braz. J. Phys. Ther..

[41] Hoshi R.A., Pastre C.M., Vanderlei L.C., Godoy M.F. (2013). Poincaré plot indexes of heart rate variability: Relationships with other nonlinear variables. Auton. Neurosci..

[42] Naranjo-Orellana J., De la Cruz-Torres B., Sarabia-Cachadiña E., De Hoyo M., Domínguez-Cobo S. (2015). Two new indexes for the assessment of autonomic balance in elite soccer players. Int. J. Sports Physiol. Perform..

[43] Cohen J. (1988). Statistical Power Analysis for the Behavioral Sciences.

[44] Goudman L., Brouns R., Linderoth B., Moens M. (2019). Effects of spinal cord stimulation on heart rate variability in patients with Failed Back Surgery Syndrome. PLoS ONE.

[45] García-Bermejo P., De la Cruz-Torres B., Naranja-Orellana J., Albornoz-Cabello M. (2017). Autonomicactivity in womenduringpercutaneousneedleelectrolysis. Eur. J. Integr. Med..

[46] Albornoz-Cabello M., Maya-Martín J. (2021). Electroestimulación, Neuromuscular y Neuromodulación.

[47] Wolff B., Burns J.W., Quartana P.J., Lofland K., Bruehl S., Chung O.Y. (2008). Pain catastrophizing, physiological indexes, and chronic pain severity: Tests of mediation and moderation models. J. Behav. Med..

[48] Abuín-Porras V., Clemente-Suárez V.J., Jaén-Crespo G., Navarro-Flores E., Pareja-Galeano H., Romero-Morales C. (2021). Effect of Physiotherapy Treatment in the Autonomic Activation and Pain Perception in Male Patients with Non-Specific Subacute Low Back Pain. J. Clin. Med..

[49] Oosterhof J., Wilder-Smith O.H., De Boo T., Oostendorp R.A.B., Crul B.J.P. (2012). The long-term outcome of transcutaneous electrical nerve stimulation in the treatment for patients with chronic pain: A randomized, placebo-controlled trial. Pain Pract..

[50] Fuentes J., Armijo-Olivo S., Funabashi M., Miciak M., Dick B., Warren S., Rashiq S., Magee D.J., Gross D.P. (2014). Enhanced therapeutic alliance modulates pain intensity and muscle pain sensitivity in patients with chronic low back pain: An experimental controlled study. Phys. Ther..

[51] Watanabe N., Reece B., Polus B.I. (2007). Effects of body position on autonomic regulation of cardiovascular function in young, healthy adults. Chiropr. Osteopat..

[52] Terkelsen A.J., Molgaard H., Hansen J., Finnerup N.B., Kroner K., Jensen T.S. (2012). Heart Rate Variability in Complex Regional Pain Syndrome during Rest and Mental and Orthostatic Stress. J. Am. Soc. Anesthesiol..

